# The association between acute brucellosis with a Guillain–Barré syndrome-like presentation: a case report

**DOI:** 10.1186/s13256-022-03740-w

**Published:** 2023-01-26

**Authors:** Leen Jamel Doya, Ibrahim Haidar, Souad Sakkour

**Affiliations:** 1 Department of Pediatrics, Tishreen University Hospital, Lattakia, Syria; 2 Faculty of Medicine, Tishreen University Hospital, Lattakia, Syria

**Keywords:** Guillain–Barré syndrome (GBS), Brucellosis

## Abstract

**Background:**

Brucellosis is a zoonotic disease caused by Gram-negative bacteria. It has variable manifestations (gastrointestinal, hepatobiliary, skeletal). Neurobrucellosis may develop at any stage of the disease (acute, subacute, and chronic phases) and affects the central and peripheral nervous systems. Acute peripheral neuropathy mimicking Guillain–Barré syndrome caused by brucellosis is rarely reported: only four cases in children were found in the literature review.

**Case presentation:**

We report a case of a 4-year-old Syrian boy who presented with fever, weakness of lower limbs, backache, and fatigue. The patient lived in a brucellosis endemic area. A physical examination including a neurological examination showed mild paresthesia and muscle weakness. He had a stiff neck with Kernig’s sign with an absence of deep tendon reflexes in the lower extremities. Proprioception in the lower extremities was impaired, but he did not have any sensory problems. Abdominal cutaneous reflexes were absent. Brucellosis and Guillain–Barré syndrome were found in laboratory investigations and on electroneurogram (ENG). The patient was treated with sulfamethoxazole + trimethoprim, rifampicin, gentamicin, and dexamethasone, with an improvement.

**Conclusion:**

This case demonstrates a rare case of brucellosis neurologic manifestation. Brucellosis should be kept in mind in all patients with acute paralysis, especially in those who live in endemic areas.

## Background

Brucellosis is a zoonotic disease caused by small intracellular aerobic Gram-negative bacilli bacteria called *Brucella* [1]. It is transmitted to humans by direct contact with an infected animal or ingestion of unpasteurized milk derivatives [2]. It is an important endemic public health problem in many countries throughout the world, including developing countries such as Turkey [3]. The most prominent symptoms are hyperthermia, fatigue, weight loss, loss of appetite, and severe pain in the joints, spine, and lower back [4]. Neurobrucellosis (NB) may develop at any stage of the disease (acute, subacute, and chronic phases) and affects the central and peripheral nervous systems. It may have widely variable manifestations, including meningoencephalitis, cranial neuropathy, brain abscess, myelitis, cerebellitis, peripheral neuropathy, and Guillain–Barré syndrome (GBS)-like presentation [5]. The prevalence of NB in children ranges from less than 1.0% to 2.2%, with a mortality rate of 0–27% and a slight predominance in males (male to female ratio of approximately 2:1) [6]. The diagnostic criteria of NB in the literature are controversial; it might be based on clinical neurological symptoms according to some authors, whereas for others, the diagnosis is based on microbiological and/or biochemical evidence from cerebrospinal fluid (CSF) [5]**.** Herein we present a case of a child with clinically and laboratory diagnosed GBS in association with acute brucellosis.

## Case presentation

A 4-year-old Syrian male was admitted to the pediatric department in Tishreen University Hospital with a 15-day history of prolonged fever measured at 38.5–39 °C at a rate of four to five times daily, partly controlled with antipyretics, and associated with fatigue, backache, headache, and lower extremities weakness. The child was treated with nonsteroidal antiinflammatory drugs (NSAIDs) without improvement and increased weakness. The patient lived in a village in an endemic area for brucellosis, with immediate contact with contaminated animals and reported consumption of unpasteurized dairy products.

In his medical history, he had suffered from prolonged fever with joint pain and fatigue a year ago, and was diagnosed with brucellosis by positive Wright serologic tests (more than 1/320) and treated orally for 45 days without completing the treatment. There was no significant medical family history.

On physical examination, his body weight was 15 kg (Standard Deviation (SD) −2.7) and his height was 98 cm (Standard Deviation (SD) M), temperature was 38.3 °C, oxygen saturation 98%, and arterial blood pressure 110/80 mmHg. He was stable and generally well without being able to stand unsupported while sitting alone.

A physical examination including a neurological examination showed mild paresthesia and muscle weakness. The muscle strength level of the arms was 4/5, the proximate muscle strength level of the legs was 2/5, and the distal muscle strength level was 2. He had a stiff neck with Kernig’s sign and deep tendon reflexes (DTRs) were absent in the lower extremities. Plantar reflexes were normal. Proprioception in the lower extremities was impaired, but he did not have any sensory problems. Abdominal cutaneous reflexes were absent and there was no disturbance in the sphincter. The spleen was palpable 2 cm below the costal margin, but the rest of the abdominal physical examination was normal. On laboratory analysis, the erythrocyte sedimentation rate (ESR) and creatine phosphokinase (CPK) were elevated. Complete blood count (CBC), C-reactive protein (CRP), renal, and liver function, glucose, and urine were normal (Table 1).

**Table 1:** Laboratory data of the case

Test	Result	Normal range	Test	Result	Normal range
WBC (10^3^/μL)	11.9	6.2–17	AST (U/L)	36	5–40
Neutrophils (%)	37	40–60	ESR (1 hour)(mm/hour)	30	0–10
Lymphocyte (%)	67	20–40	Glucose (mg/dL)	100	70 -100
Hb (g/dL)	11.5	11–13	CPK (U/L)	852	21–230
MCV (fl)	66	70–85	Urea (mg/dL)	33	15–36
PLT (10^3^/μL)	322	150–450	Creatinine (mmol/L)	0.2	0.5–1.3
CRP (mg/dL)	3	< 5	K (mmol/L)	3.9	3–4.5
ALT (U/L)	39	7–55	Na (mmol/L)	133	135–145

*WBC* white blood cell, *HB* hemoglobin, *MCV* mean corpuscular volume, *RDW* red cell distribution width, *PLT* platelets, *CRP* C-reactive protein, *ALT* alanine aminotransferase, *AST* aspartate aminotransferase, *ESR* erythrocyte sedimentation rate, *CPK* creatine phosphokinase

The Widal test was negative for typhoid (O: 1/80; H: 1/80), while the Wright test was positive (more than 1/320). We could not identify the titers of the antibodies because there were not available in our country. CSF analysis showed albumino-cytological dissociation [white blood cell counts (lymphocytes) of 10 cells/cubic millimeter, CSF glucose 57.47 mg/dL (normal value: 50–80 mg/dl); the corresponding blood glucose was 169 mg/dL (normal value: 60–100 mg/dL), and a protein level of 217.4 mg/dL (normal value: 15–45 mg/dL)]. The CSF culture was negative.

Abdominal ultrasound showed mild splenomegaly without evidence of space-occupying lesions. A computed tomography scan (CT), and subsequent magnetic resonance imaging (MRI) of the brain and whole spine were unremarkable. Funduscopic examination and abdominal ultrasound were normal.

A nerve conduction study (NCS) and needle electromyography (EMG) revealed evidence of axonal and demyelinating polyneuropathy. The velocity of sensory nerve transmission was decreased in the legs. The neuromuscular transmission was normal. Based on previous data, the patient was diagnosed with an association between acute brucellosis and a GBS-like presentation.

Due to the lack of availability of an intravenous immune globulin (IVIG) and plasma exchange in our hospital, the patient was treated with sulfamethoxazole + trimethoprim (10 mg/kg/day), rifampicin (15 mg/kg/day), gentamicin (5 mg/kg/day), and dexamethasone (0.5 mg/kg/day).

After 7 days of treatment, the child’s symptoms improved (fever, headache, and backache) with the continued inability to walk and pain in the lower extremities, and the absence of tendon reflexes in the lower extremities.

After 15 days, the patient was discharged with instructions to follow the treatment of sulfamethoxazole + trimethoprim (10 mg/kg/day), rifampicin (15 mg/kg/day) for 6 months, and vitamins.

The child improved gradually after discharge. The muscle weakness in the lower extremities improved, and he walked with support a month after discharge. He walked without support 2 months after discharge. Currently, the child is in good condition with a normal movement and growth rate.

## Discussion and conclusion

NB was first reported in 1896 by Hughes. The mechanism of *Brucella* infection that triggers an immune response against autoantigens and causes polyradiculopathy during the acute phase of illness is unknown. It may be due to immunologic and inflammatory reactions [7]. In an experimental animal study, the ganglioside-like molecules expressed on the surface of *Brucella* induced autoantibodies against myelin gangliosides, resulting in acute paralysis and GBS signs [6].

The neurologic symptoms are rarely the initial complaint. NB has usually nonspecific manifestations (headache, neck stiffness, back pain, spastic paraparesis, seizures especially if there is underlying cerebral venous sinus thrombosis, and acute meningitis or meningoencephalitis) that may imitate various pathologies [8].

NS is usually diagnosed by the history of ingesting milk or dairy products with any one of the following criteria: (1) lack of typical signs of other neurological diseases; (2) isolation of *Brucella* species from the CSF and/or presence of anti-Brucella antibodies in CSF; (3) the presence of lymphocytosis, increased protein, and decreased glucose levels in CSF; (4) diagnostic findings in cranial MRI or CT; (5) and the response of symptoms with appropriate treatment (a drop in the protein and lymphocyte count in the CSF) [9].

Most cases of *Brucella* meningitis (91%) are lymphocytic pleocytosis in CSF with elevated protein and normal or low glucose. In NB that affects the cerebellum, there is elevated protein in CSF with no leukocytosis. CNS culture in NB is frequently sterile; only 30% of patients with NB had positive blood culture and 14% had positive CSF culture [8].

Our patient had movement impairment (weakness and muscular pain in the lower extremities with absence of deep tendon reflexes) with a history of diagnosis with brucellosis, ingesting unpasteurized milk products, living in a village in an endemic area for brucellosis, lack of typical signs of a known other neurological disease, and a positive serological test (Wright reaction; 1/320), which without being able to perform the titers of the antibodies, were essential data for suspicion of brucellosis. The diagnosis of GBS was confirmed with CSF (albumino-cytological dissociation) and electrophysiologically (multiple demyelinating polyneuropathy). The elevated protein and lymphocytic pleocytosis may suggest coexisting meningoencephalitis.

There is no consensus in the medical literature about the basic treatment of NB, but there is an agreement on the use of several antibiotics for reducing relapses or avoiding failure. The commonly used treatment for NB (GBS with brucellosis) is the combination of two or three appropriate antibiotics (doxycycline, rifampicin, trimethoprim–sulfamethoxazole, streptomycin or ceftriaxone) for long periods (ranging from 2 to 15 months, but at least 6 months is recommended) with intravenous immunoglobulin or plasma exchange sessions [9]. The recommendation of the American Academy of Pediatrics (AAP) is the use of gentamicin initially for 7–14 days in addition to rifampin and tetracycline/trimethoprim–sulfamethoxazole for at least 6 weeks to 7 months according to the clinical response and return of CSF components to normal [9].

The indication of corticosteroids in the therapeutic management of brucellosis in combination with antibiotics is not well codified. Some authors indicate corticosteroid therapy systematically for NB, while others prescribe it in case of severe forms. Corticosteroid therapy is recommended to reduce inflammation and improve neurologic outcomes in patients with NB [10]. Based on the economic condition in our country, the patient was treated with broad-spectrum antibiotics (sulfamethoxazole + trimethoprim) and dexamethasone.

NB is associated with some complications such as cranial nerve involvement (sixth, seventh, and eighth) (19%), polyneuropathy/radiculopathy (7%), depression (5%), paraplegia (4%), stroke, and abscess formation (3%). Meningitis and meningoencephalitis are the most common complications of NB in children [10]. The prognosis of NB with meningitis is usually good, but consequences are more common in those with spinal cord involvement [8].

In the medical literature review, there is insufficient data about the relationship between *Brucella* spp. and GBS in children. We could find only a few case reports about GBS with *Brucella* infection in childhood (Table 2).

**Table 2:** The literature on cases of GBS with *Brucella* infection in childhood

Author/date	The patient	Manifestation	Diagnosis	Treatment
Al Essa Ya 1996	14-year-old girl with GBS associated with *Brucella* infection	Acute progressive ascending symmetric weakness, protracted paroxysms of severe hypertension	Coombs Wright titration Nerve conduction studies	Rifampicin + doxycycline Plasma exchange sessions Partial recovery after 2 months
Namiduru 2003	14-year-old girl with GBS associated with brucellosis	Acute progressive ascending symmetric weakness	CSF culture for *Brucella* Wright agglutination test Nerve conduction studies	Rifampicin + cotrimoxazole Plasma exchange sessions
Barzegar 2009	9-year-old boy with GBS associated with brucellosis	Progressive weakness in the lower extremities paresthesia	Wright agglutination test Mercaptoethanol titer negative Nerve conduction studies	Plasmapheresis in four sessions every other day
Onur Balci 2013	11-year-old female with brucellosis diagnosed with GBS	Fever, pain while walking, pain on knees and elbows, pain, and numbness on hips and legs for 3 weeks	*Brucella* agglutination Test *Brucella* IgG was positive Positive blood culture Nerve conduction studies	Rifampicin + streptomycin + doxycycline Intravenous immunoglobulin for 5 days

This case demonstrates a rare case of brucellosis neurologic manifestation. Brucellosis should be kept in mind in all patients with acute paralysis and GBS-like signs, especially in those who live in endemic areas

## Data Availability

All data generated or analyzed during this study are included in this published article.
